# The Role of Social Contagion in Initiating Non-Suicidal Self-Injury in Schizophrenia: A Case Report

**DOI:** 10.62641/aep.v54i3.2190

**Published:** 2026-06-15

**Authors:** Jonathan Shaw, Peter Bota, Charles Lai, Aidan Yong, Justin Cheng, Tina Allee

**Affiliations:** ^1^School of Medicine, California University of Science and Medicine, Colton, CA 92324, USA; ^2^Psychiatry, Brain Health Solutions, Costa Mesa, CA 92626, USA

**Keywords:** schizophrenia, self-injurious behavior, substance-related disorders, ligation, psychotic disorders

## Abstract

Non-suicidal self-injury (NSSI) is a highly prevalent maladaptive behavior with studies indicating that it can affect between 40–80% of individuals with schizophrenia spectrum disorder. NSSI can arise during any point during the course of schizophrenia spectrum disorder and has the potential to suddenly manifest as major-self mutilation (MSM), a particularly severe and catastrophic form of NSSI. Although the motivation for NSSI/MSM varies by patient, the literature indicates that there are factors associated with these behaviors such as first-episode psychosis, command hallucinations, and past self-harming episodes. In this case report, the authors discuss a rare case of a 25-year-old male with schizophrenia who presented with ligature of multiple digits on both hands. The authors examine this rare presentation and how it is suspected to be linked to a similar case in nearby Los Angeles county through social contagion. Through this case, the authors aim to highlight the prevalence of NSSI, the potential for NSSI to transform to MSM, and the need for ongoing screening for these behaviors in those with psychotic disorder diagnoses.

## Introduction

Schizophrenia is a psychotic disorder which affects a significant proportion of individuals receiving high-level psychiatric services, with an annual incidence rate of 0.2–0.4 per 1000 and a median lifetime prevalence of 4.00 per 1000, though some studies indicate the lifetime risk of schizophrenia differ slightly between the sexes (1.93% in males and 1.56% in females) [1, 2, 3]. Common symptoms of schizophrenia include delusions, hallucinations, social isolation, and disorganized behavior [4, 5, 6]. Individuals with schizophrenia also have higher lifetime rates of suicide attempts and non-suicidal self-injury (NSSI), defined as deliberate self-harm without suicidal intent, at 26.8% and 43.6%, respectively [7, 8]. Psychotic disorders are strongly associated with major self-mutilation (MSM), a severe form of NSSI involving loss of organ or appendage function [9, 10]. Most individuals presenting with MSM (75.6%) have a psychotic disorder, with 83.2% of them specifically diagnosed with the schizophrenia spectrum psychosis [9, 10].

Depending on the method of self-harm, NSSI carries risks including accidental death, infection, and permanent injury or scarring [11, 12]. Those with schizophrenia also face barriers to care such as low health literacy, comorbid substance use, and limited access to services, all of which are associated with increased odds of premature mortality [13, 14, 15, 16]. However, it should be noted a direct causal relationship between premature mortality and these variables is not explicitly established by the literature, instead these are factors which may indirectly contribute to this observed trend. Common NSSI behaviors in schizophrenia include cutting (37.5%), hair pulling (11.1%), and scratching (11.1%), while MSM is rare and typically associated with severe mental illness and often results in permanent organ damage or loss of function [8, 17].

This case report describes a 25-year-old male with schizophrenia and stimulant use disorder who presented with multiple digit infections after ligation, representing the second reported U.S. case; the first having occurred in Los Angeles County [18]. The patient attributed his NSSI behaviors to social contagion, defined as the spread of behaviors through interpersonal contact, and similarity to the prior case suggests possible influence [19].

This case highlights the potential for individuals with schizophrenia to present with chronic, but undetected NSSI that may escalate during periods of psychiatric decompensation. It also emphasizes the need for ongoing NSSI and suicide-risk screening in this population and provides an opportunity for clinicians and researchers to track this rare presentation of NSSI that may be influenced by social contagion in California.

## Case Report

JD is a 25-year-old caucasian male who was brought to the psychiatric emergency treatment service of a community hospital by community behavioral assessment team and police on a 72-hour psychiatric hold (Lanterman-Petris-Short Act 5150) for danger to self in early 2025 after endorsing suicidal ideation with a plan to slit his throat. However, upon arrival, JD states he does not have an active plan for committing suicide anymore. His psychiatric history includes schizophrenia, conduct disorder, methamphetamine abuse, cannabis abuse, and severe recurrent major depression with psychotic features. Most diagnoses date to 2019, when he experienced his first psychiatric hospitalization at a Los Angeles County psychiatric hospital. JD reported daily methamphetamine use beginning in 2016 and spending significant time unhoused. Chart review revealed incomplete records but indicated two additional psychiatric hospitalizations in 2024 in the Inland Empire. He also presented to emergency departments four times in 2024 and once in early 2025, primarily seeking help for substance use and reporting suicidal ideation without an active plan to commit suicide. Previous medication trials included olanzapine, quetiapine, risperidone, aripiprazole, hydroxyzine, fluoxetine, and valproate. At the time of this encounter, his outpatient medications were fluoxetine 20 mg daily, olanzapine 20 mg orally disintegrating tablet daily, and trazodone 50 mg nightly.

While in the emergency psychiatric unit, JD appeared disheveled, restless, and guarded while also reporting auditory hallucinations consisting of voices saying “yes and no.” JD endorsed recent substance abuse, including crystal methamphetamine use approximately four hours prior to presentation and alcohol use two days prior. Soon after completing intake procedures, JD demonstrated verbal agitation, hostility towards hospital staff, and exhibited unpredictable, non-redirectable behavior, including delusional statements such as the belief that he was an alien/extra-terrestrial. Due to his psychotic symptoms and risk of danger to others, JD received emergency intramuscular medications (diphenhydramine (67890BC, Hospira Inc, Lake Forest, Illinois, USA) 50 mg, haloperidol (25D031A, Fresenius Kabi, Grand Island, NY, USA) 5 mg, and lorazepam (45231DF, Hospira Inc, McPherson, Kansas, USA) 2 mg), after which JD became calm and fell asleep.

On initial evaluation by a psychiatrist after he woke up, JD demonstrated a mild tremor and clarified that he had drunk a 750 mL bottle of tequila two days prior. He also revealed that he had eloped from his board and care one-week prior to presenting to the psychiatric emergency department so he could use methamphetamine with a friend. JD then received a one-time dose of chlordiazepoxide (023J5431, Chartwell RX LLC, Congers, NY, USA) 25 mg and was started on gabapentin (RUPJ382A, Viatris, Greensboro, NC, USA) 300 mg three times daily for alcohol withdrawal prophylaxis. An overnight internal medicine consult was placed due to worsening pain and swelling of JD’s right hand, with a recommendation to transfer JD to the medical emergency department for further imaging and an Orthopedic/Hand surgery evaluation to assess the need for incision and drainage.

In the medical emergency department, JD reported that he had been tying hair ties tightly around his fingers, “to see how long I could keep them on,” and had left them in place for three days. During later interviews by the consultation liaison team, JD initially refused to speak about the ligation or endorsed a delusional conviction that this was, “the right thing to do,” but later admitted that he had, while unhoused in Los Angeles County, “seen someone else do this, thought it was cool, and started doing it.” JD had ligated two fingers on each hand (four total) and stated that he had engaged in this behavior intermittently for the past four years, though this was the first instance of severe injury due to placing the bands too tightly. JD also reported that the cuts on his right third and fourth fingers may have been caused by tying his bandana too tightly, though this mechanism did not appear to match the observed injuries and the authors believe that all of JD’s hand injuries stem from auto-ligation using hair ties.

Examination revealed multiple wounds on JD’s right hand with purulent drainage, edema, and marked tenderness, most pronounced in the right fourth finger. Radial pulse was 2+, and sensation remained intact. Orthopedic surgery was consulted, and JD was admitted to internal medicine for suppurative flexor tenosynovitis of the right fourth finger and right hand cellulitis. JD underwent bedside incision and drainage in the medical emergency department, followed by operative washout two days later by orthopedic surgery. Both procedures were well tolerated without complications. Blood cultures were negative, but wound cultures from both procedures grew Streptococcus pyogenes. JD also received a Tdap booster in the medical emergency department.

JD was initially treated with two days of intravenous clindamycin (RUPJ284A, Viatris, Greensboro, NC, USA) 600 mg and ceftriaxone (S21045B, Sagent Pharmaceuticals, Plattsburgh, NY, USA) 2 g every 24 hours in the emergency department, followed by a six-day course of ampicillin/sulbactam (GP31245, Pfizer, Kalamazoo, Michigan, USA). He was then transitioned to oral amoxicillin/clavulanate (US23045, USAntibiotics LLC, Bristol, Tennessee, USA) 875/125 mg every 12 hours to complete a three-week antibiotic course from the date of operative washout. He tolerated treatment well, with resolution of leukocytosis and no fevers prior to discharge. During vancomycin (V210457, Hikma Pharmaceuticals USA Inc, Bedford, Ohio, USA) infusion in the emergency department, JD developed distal erythema and swelling below the peripheral IV site that briefly extended to the elbow and distal humerus but improved with limb elevation. The infusion was stopped, and an allergy to vancomycin-related (V210457, Hikma Pharmaceuticals USA Inc, Bedford, Ohio, USA) antibiotics was added to his chart.

Psychiatrically, JD’s 72-hour hold (5150) was extended to a 14-day hold (5250) during admission. He was restarted on his psychiatric medications and showed consistent symptomatic improvement throughout hospitalization. Psychotherapy was offered but declined. Two days after restarting his medications, JD developed excessive daytime sedation, and his olanzapine (B312457, Eli Lilly and Company, Kinsale, Ireland) dose was reduced to 10 mg daily. No further adverse effects were observed, and he continued to respond well to treatment. JD expressed embarrassment about his substance use relapse and prior absent without official leave (AWOL) behavior and made future-oriented statements about wanting to become sober. A bedside sitter observed no suicidal or homicidal ideation and no evidence of auditory or visual hallucinations during the remainder of his stay.

After evaluation by physical and occupational therapy and clearance for discharge, JD returned to his board-and-care facility after an 11-day hospitalization with a 30-day supply of his psychiatric medications and the remainder of his antibiotic course. At discharge, his psychiatric medications included olanzapine (B312457, Eli Lilly and Company, Kinsale, Ireland) 10 mg nightly, fluoxetine (D045789, Eli Lilly and Company, Indianapolis, Indiana, USA) 20 mg daily, and trazodone (K190872, Aurobindo Pharma, Medchal, India) 50 mg nightly. He remained connected with the same outpatient psychiatrist and case manager that he had prior to admission. A summary of JD’s clinical course can be seen in Table 1.

**Table 1.** **Hospital course**.

Hospital day	Time	Clinical event
Day 1	17:52	JD presents to the emergency treatment service on a 72-hour psychiatric hold for suicidal ideation with a plan to slit his throat with a knife. JD states he has suicidal ideation with no active plan while in the emergency treatment service.
Day 1	19:44	JD demonstrates verbal agitation and hostility towards staff. Intramuscular 50 mg diphenhydramine (67890BC, Hospira Inc, Lake Forest, Illinois, USA), 5 mg haloperidol (25D031A, Fresenius Kabi, Grand Island, NY, USA), and 2 mg lorazepam (45231DF, Hospira Inc, McPherson, Kansas, USA) is administered.
Day 2	4:37	JD wakes up and is amenable to interview with overnight psychiatrist. Mild tremor is noticed in both hands. JD states he drank 750 mL of tequila two days prior. Internal medicine consult placed due to hand wounds.
Day 2	6:12	Internal medicine team recommends transfer to medical emergency department for further imaging and an orthopedic/hand surgery evaluation for incision and drainage for hand wounds.
Day 2	8:22	JD arrives at medical emergency department. JD states he had ligated his fingers with hair ties and left them there for 3 days. JD states he began doing this four years ago after observing someone in Los Angeles county auto-ligating.
Day 2	8:50	JD is started on the following antibiotics:
		2-day course of IV clindamycin (RUPJ284A, Viatris, Greensboro, NC, USA) 600 mg
		2-day course of IV ceftriaxone (S21045B, Sagent Pharmaceuticals, Plattsburgh, NY, USA) 2g every 24-hours
		JD was also started on IV vancomycin (V210457, Hikma Pharmaceuticals USA Inc, Bedford, Ohio, USA) but was noted to have erythema and swelling of his peripheral IV site which improved with limb elevation. The vancomycin (V210457, Hikma Pharmaceuticals USA Inc, Bedford, Ohio, USA) was immediately halted and an allergy to vancomycin-related antibiotics was added to JD’s chart.
Day 2	10:35	Orthopedic consult arrives and, upon reviewing imaging, determines a bed-side incision and drainage is indicated. The procedure is tolerated well by the patient and samples are taken for wound culture. Orthopedics plans for a potential follow-up procedure in the operating room. JD admitted to internal medicine.
Day 2	11:20	Psychiatry consultation-liaison team transitions JD from a 72-hour psychiatric hold to a 14-day psychiatric hold given his medical and psychiatric complaints. JD is restarted on his home medications of fluoxetine (D045789, Eli Lilly and Company, Kinsale, Ireland) 20 mg daily, olanzapine (B312457, Eli Lilly and Company, Kinsale, Ireland) 20 mg daily, and trazodone (K190872, Aurobindo Pharma, Medchal, India) 50 mg nightly.
Day 4		JD is taken to the operating room for another incision and drainage by orthopedic surgery, tolerating the procedure well. Upon completing his ceftriaxone (S21045B, Sagent Pharmaceuticals, Plattsburgh, NY, USA) and clindamycin (RUPJ284A, Viatris, Greensboro, NC, USA) courses, JD is started on oral amoxicillin/clavulanate (US23045, USAntibiotics LLC, Bristol, Tennessee, USA) 875/125 mg every 12 hours for 19 days (to complete a 3-week antibiotic course). Wound cultures grew *streptococcus pyogenes*.
Day 4		Excessive daytime noted with JD during psychiatric rounds, oral olanzapine (B312457, Eli Lilly and Company, Kinsale, Ireland) tapered from 20 mg to 10 mg daily. No further adverse effects from his medication are noted.
Days 5–10		JD’s condition gradually and consistently improves both physically and psychiatrically. JD’s leukocytosis resolves and he begins to make future-oriented statements about wishing to attend substance rehabilitation programs and to become sober.
Day 11		JD is noted to have already been connected with outpatient psychiatric and already has a case manager. JD is discharged to his original board and care.

## Discussion

This case report describes a 25-year-old male whose chronic and recurrent NSSI nearly progressed to MSM. During his admission, JD provided multiple explanations for his NSSI, though psychiatric decompensation during a one-week elopement from his board-and-care facility may have contributed to the escalation in severity. Several factors may have influenced his behavior, including psychosis related to schizophrenia and active methamphetamine use, as well as suicidality potentially associated with polysubstance use or recurrent severe major depression. However, no anxiety or depressive symptoms were observed during this admission, making recurrent major depression with psychotic features a less likely contributor. Instead, the authors believe that a combination of medication non-compliance and methamphetamine use led to JD’s psychiatric decompensation and resulted in a sudden increase in his NSSI severity.

Interestingly, JD reported that his NSSI began after observing another individual performing finger ligation while he was living intermittently on the streets and in institutions in Los Angeles County, suggesting social contagion as a possible initiating factor [19]. NSSI has been shown to spread through social contagion, particularly among young adults, individuals with psychiatric conditions, and those in institutional settings, all of which applied to JD [20]. The social contagion aspect is notable because JD’s attempted MSM is highly unusual. MSM typically occurs violently and impulsively, most often as auto-enucleation, self-castration (Klingsor syndrome), and less commonly limb amputation, usually proximal to the wrist [10, 21]. The same psychiatric team later evaluated another unhoused Los Angeles patient with polysubstance use and psychosis who presented with finger ligation. Both cases closely resemble a 2021 Los Angeles County case [18]. At the time of the 2021 Los Angeles County case report, only one prior case of intentional banding of multiple digits in a psychiatric context had been reported, and none in the United States [18, 22].

However, JD’s reason for adopting auto-ligation NSSI cannot be confirmed, as this behavior was not observed by family or caregivers over several years. Individuals with schizophrenia are more likely to confabulate details when recalling events than controls, and such confabulation is associated with positive symptoms like formal thought disorder and delusions [23, 24]. This lack of collateral information, along with potential confabulation amid JD’s psychiatric decompensation from medication nonadherence and stimulant use, prevents the authors from identifying social contagion as the definitive cause of his NSSI, despite supporting circumstantial evidence.

Regardless of the cause of these NSSI, this case highlights the need to monitor individuals with schizophrenia for NSSI and suicidal ideation. Although JD’s NSSI presentation was unusual, his treatment was routine, including antibiotics, surgical care, and the resumption of his psychiatric medications with minor dose adjustments. However, his report of engaging in the same NSSI for four years without prior documentation is concerning. NSSI is relatively common in schizophrenia spectrum disorders, with a lifetime prevalence of 32.6% (95% CI: 13.3%; 60.4%), and can occur at any stage, though MSM is associated with first-episode and prolonged untreated psychosis [9, 10, 25, 26].

Given these risks, some studies recommend screening for NSSI and suicidal ideation at every clinical encounter with patients with schizophrenia, especially before discharge. The Patient Health Questionnaire-9 (PHQ-9) reliably predicts suicidality risk within 90 days, and the Stanley-Brown Safety Plan Intervention may also be employed [27, 28]. When social contagion may contribute to NSSI, clinicians should also provide individualized psychoeducation [19].

Although access to outside records was limited, available documentation did not indicate a prior history of NSSI in JD. Fortunately, JD was treated before severe complications developed. Increased depression scores are strongly associated with suicidality (Hazard Ratio (HR) 3.5) [29]. Similarly, substance use disorders further elevate risk, with amphetamine use associated with twice the odds of psychosis and a 4.4-fold increase in suicidality, while alcohol use disorder comorbid with another substance use disorder carries a 1.96 odds ratio for suicide attempts [30, 31]. Clinicians should remain vigilant for NSSI and suicide risk, using tools like the PHQ-9 and the Stanley-Brown Safety Planning Intervention in outpatient and discharge settings [27, 28].

This case report is primarily limited by the multifactorial and self-reported nature of JD’s behaviors. Chart review indicated that JD carried several psychiatric diagnoses that could have contributed to his NSSI. However, he consistently reported that he adopted the unusual behavior of ligating multiple digits after observing someone perform the same act while he was in Los Angeles. While the possibility that JD was influenced by social contagion related to the previously reported Los Angeles case remains speculative, the circumstantial details he provided lend some support to this interpretation. Additionally, JD did not provide a written patient perspective for the authors despite providing written informed consent for the publication of this case report.

## Conclusions

As this case shows, NSSI can remain undetected for extended periods before worsening and potentially progressing to MSM. In JD’s case, the increased severity was likely driven by medication nonadherence and methamphetamine use causing psychiatric decompensation. His repeated reports of adopting auto-ligation after observing it in Los Angeles County suggest social contagion as a key initiating factor, though this could potentially be a delusion or confabulation. Despite this, clinicians should still consider the influence of social context on behavior and provide tailored psychoeducation to counter the adoption of harmful behaviors.

## Availability of Data and Materials

Not applicable.

## Supplementary Material

Supplementary material associated with this article can be found, in the online version, at https://doi.org/10.62641/aep.v54i3.2190.
